# CCD-Based Skinning Injury Recognition on Potato Tubers (*Solanum tuberosum* L.): A Comparison between Visible and Biospeckle Imaging

**DOI:** 10.3390/s16101734

**Published:** 2016-10-18

**Authors:** Yingwang Gao, Jinfeng Geng, Xiuqin Rao, Yibin Ying

**Affiliations:** 1 College of Biosystems Engineering and Food Science, Zhejiang University, Hangzhou 310058, China; huneagler@163.com (Y.G.); kuailehongliu@126.com (J.G.); ybying@zju.edu.cn (Y.Y.); 2 Key Laboratory of Equipment and Informatization in Environment Controlled Agriculture, Ministry of Agriculture, Zhejiang University, Hangzhou 310058, China

**Keywords:** skinning injury, recognition, potato, visible imaging, biospeckle imaging

## Abstract

Skinning injury on potato tubers is a kind of superficial wound that is generally inflicted by mechanical forces during harvest and postharvest handling operations. Though skinning injury is pervasive and obstructive, its detection is very limited. This study attempted to identify injured skin using two CCD (Charge Coupled Device) sensor-based machine vision technologies, i.e., visible imaging and biospeckle imaging. The identification of skinning injury was realized via exploiting features extracted from varied ROIs (Region of Interests). The features extracted from visible images were pixel-wise color and texture features, while region-wise BA (Biospeckle Activity) was calculated from biospeckle imaging. In addition, the calculation of BA using varied numbers of speckle patterns were compared. Finally, extracted features were implemented into classifiers of LS-SVM (Least Square Support Vector Machine) and BLR (Binary Logistic Regression), respectively. Results showed that color features performed better than texture features in classifying sound skin and injured skin, especially for injured skin stored no less than 1 day, with the average classification accuracy of 90%. Image capturing and processing efficiency can be speeded up in biospeckle imaging, with captured 512 frames reduced to 125 frames. Classification results obtained based on the feature of BA were acceptable for early skinning injury stored within 1 day, with the accuracy of 88.10%. It is concluded that skinning injury can be recognized by visible and biospeckle imaging during different stages. Visible imaging has the aptitude in recognizing stale skinning injury, while fresh injury can be discriminated by biospeckle imaging.

## 1. Introduction

Potato is now regarded as the fourth most important food crop around the world after wheat, rice and maize (corn), due to its great yield production and high nutritive value [1]. However, quality and nutrition of potato tubers can be adversely affected by mechanical damages, which happen frequently during the handling chain from harvest, storage, and transport to packaging. Skinning injury is related to excoriation of potato skin. In fact, potato periderm is composed of three cell layers: phellem, phellogen and phelloderm [2]. Among them, well-organized suberized cells constitute phellem, which is referred to as skin. According to Lulai [3], the resistance to skinning injury is determined by phellem (skin) tensile-related fractures and phellogen shear-related fractures, where the force required for fracture of the phellogen cell walls is dominant. Skinning injury is one kind of superficial damage on potatoes that can result in the fracture of phellogen cell walls of the native periderm and loss of the protective layer of phellem cells [4]. Skinning injury not only results in the discoloration of the wounded area, but also provides sites for infection by pathogens [5], and it can increase water loss and wastage during storage [6]. It has been reported as a common, persistent and costly problem of the potato industry [4].

Nowadays, machine vision technology has been attracting more attention in scientific inspection for quality and safety of a variety of food and agricultural products [7]. Visible imaging can be regarded as one traditional machine vision technology working in the range of visible wavelengths (380–780 nm). Color and texture are two sensory quality attributes that have been frequently used in the external quality inspecting and grading. Containing the basic visual information in the images corresponding to human vision, color is viewed as the most elementary information that is stored in pixels [8]. The aim of texture analysis is to try to discriminate different patterns in images by obtaining the variance of intensity values across pixels or by extracting the dependency of intensity values between pixels and their neighboring pixels [9,10]. Color and texture features have been used on potato external quality inspection. Marique et al. [11] developed a procedure to process and segment potato images using Kohonen’s self-organizing map (SOM). RGB values of each pixel were fed into the model to discriminate between pixels associated with either healthy or bruised parts of potato tubers. They found that bruises that were very dissimilar in appearance were correctly identified, and some particular defects such as green spots could be located as well. In a broad scale, Ebrahimi et al. [12] believed that the difference between red and green components of RGB space was effective in greening detection in potatoes. Color and texture features are often combined to improve detection. Barnes et al. [13] used an adaptive boosting algorithm (AdaBoost) to discriminate between blemishes and non-blemishes based on both the color and texture features of the region surrounding a given pixel. To detect external defects on potatoes, Moallem et al. [14] extracted RGB components of each pixel and co-occurrence texture features from the grey level component of color-space images to implement into several supervised classifiers. Results showed that SVM (Support Vector Machine) represented a higher performance for potato defect detection. Based on the above research, color and texture features were extracted and implemented into LS-SVM (Least Square Support Vector Machine) to recognize skinning injury.

The biospeckle imaging technique is an emerging non-destructive method for the evaluation of vitality of biomaterials, which is based on the biospeckle phenomenon when the biomaterial is illuminated by coherent light. The backscattered light displays granular patterns, i.e., presenting randomly distributed, time-dependent light and dark spots on CCD (Charge Coupled Device) sensor. Reflecting surface information and particle movement at the cellular/sub-cellular level, it has found its way into the applications in various agro-products/food detection areas [15,16,17,18]. Previous study in our laboratory also confirmed the feasibility of this non-destructive method in defect and stem/calyx discrimination [19]. Zdunek et al. [15] believed that light propagation and Biospeckle Activity (BA) could be affected since red laser (670 nm) is absorbed by chlorophyll. They investigated the potential interrelationships between the BA and chlorophyll content in apples. Results showed that BA linearly decreased with increasing chlorophyll content. Alves et al. [17] studied the correlation between particular phenomena (moisture content, respiration rate, water activity, and mass loss changes) and the activity observed in fresh-cut carrots. They concluded that it was viable to monitor the respiration process in fresh-cut carrots and assign a spectral signature to their water content and respiration. Arefi et al. [20] carried out research to recognize mealy from non-mealy apples via developing classification models based on biospeckle imaging. Results showed that BA was higher for fresh apples in comparison with semi-mealy and mealy samples. Skinning injury results in compositional and structural changes in wounded area, which may serve as the foundation for BA monitoring and skinning injury classification.

Though several researches have been conducted to uncover the mechanism of induction and regulation of skinning injury [21,22,23], limited progress has been made in skinning injury detection to our knowledge. The objective of this research was attempting to identify skinning injury on potatoes using two CCD-based methods, i.e., visible imaging and biospeckle imaging. Pixel-wise features including color and texture were derived from visible imaging, while the calculation of region-wise feature of BA was described in detail based on biospeckle imaging. Pixel- and region-wise features were implemented into LS-SVM and BLR (Binary Logistic Regression) to conduct classification, respectively. Finally, these two methods were compared in recognizing skinning injury.

## 2. Materials and Methods

### 2.1. Sample Preparation

**Figure 1:**
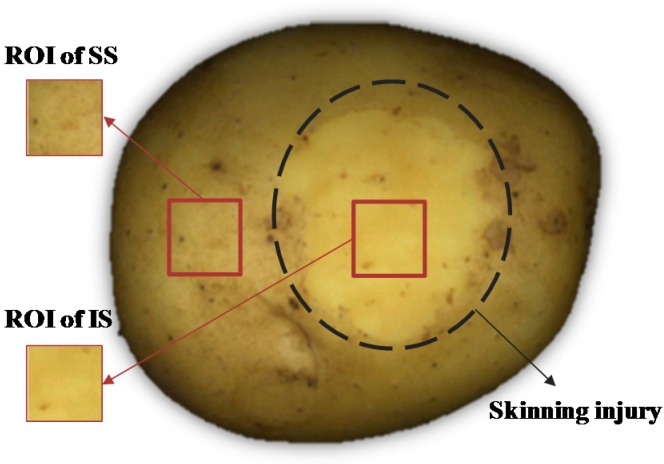
Representative image of skinning injury on potato (SS: sound skin; IS: injured skin).

Potato tubers of the cultivar “*Favorite*” (a popular cultivar in China) were purchased from local whole-sale vegetable market in Hangzhou, China. The samples (harvested in April, 2015) were bought and transported to the lab in May 2015; 120 of which were selected for experiments based on the criteria that they have similar shapes and are free from visible blemishes. All of the selected samples were used for visible imaging, while 80 of them were then used for biospeckle imaging subsequently. Samples were divided into four groups respectively, with 20 and 30 samples for each group. Skinning injury was created with a steel rod scratching the potato surface to carry out simulation in lab. Then, all samples were stored in the environment of 26 ± 2 °C and 72% ± 16% RH for up to 7 days. RGB images of the injured samples were taken after 1 h, 12 h, 1 d, 3 d and 7 d. Biospeckle images were taken immediately after visible imaging, the corresponding image capturing intervals were chosen as 1 h, 1 d, 3 d, 5 d and 7 d, along with images of sound skin. Image of a representative skinning injured potato is shown in Figure 1.

### 2.2. Image Acquisition Systems

#### 2.2.1. Visible Imaging

**Figure 2:**
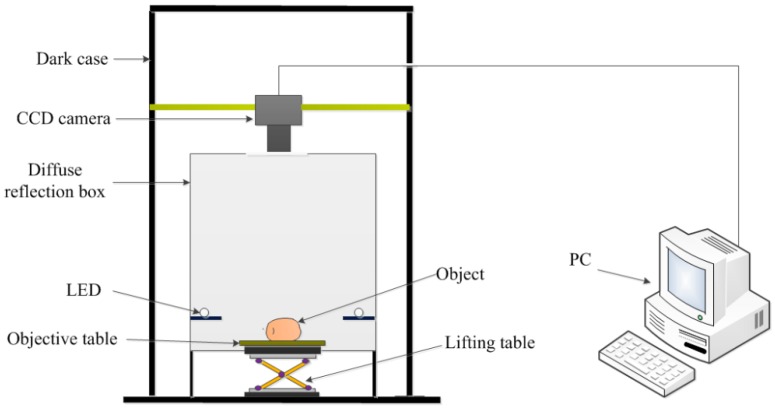
Experimental setup for visible imaging.

The experimental setup used for visible imaging is shown in Figure 2. The system consisted of a dark case to diminish the influence of ambient illumination, CCD camera (DVP-30GC03E; The Imaging Source, designed in Germany), LED lamps (18 W; Langtuo Inc. Hangzhou, China), an objective table and a lifting table, a diffuse reflection box, which is made of Teflon to make sure the incident light on the objects soft and even, and a PC. The CCD sensor is ICX445AQA, with 1.25 M effective pixels. The number of active pixels is 1280 (Horizontal) × 960 (Vertical). It bears the advantages of high resolution, high sensitivity, low dark current and low smear. It is important to make sure the injured area is in the middle of the field of view when capturing images, and accounts for about 25% of the whole captured image.

#### 2.2.2. Biospeckle Imaging

**Figure 3:**
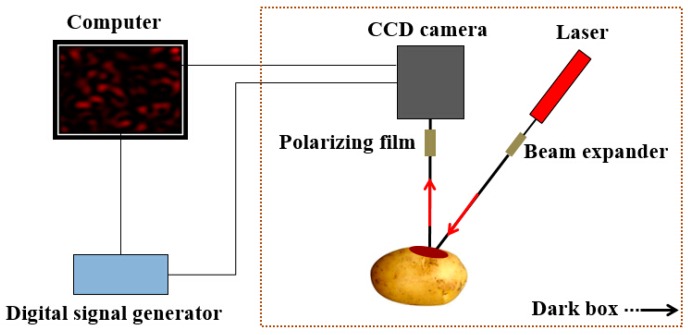
Experimental setup for biospeckle imaging.

The experimental setup used for biospeckle imaging technique is shown in Figure 3, which was adjusted from previous study in our laboratory [19]. The system consisted of a low power semiconductor laser (LSR635ML-50, 635 nm, 50 mW; Lasever Inc., Ningbo, China), with a beam expander (BEST-635-29, 20X, 20 mm; Sintec Optronics Inc., Wuhan, China) to illuminate the sample. Biospeckle was recorded by a CCD camera (DVP-20GC02E; The Imaging Source Europe GmbH, designed in Germany); with a polarizing film (Beijing Optical Century Instrument Co., Ltd., Beijing, China) to reduce illumination intensity. The specification of the CCD sensor is ICX274AQ, which has 2.01 M effective pixels. The recording pixels are 1600 (Horizontal) × 1200 (Vertical). It has high sensitivity and low dark current. Speckle images with 512 × 512 pixels (20 × 20 mm on sample surface) were cropped from original images. A temporal sequence of 512 dynamic speckle frames was recorded for one shoot with the help of a digital signal generator (SPF120; Sample Technologies, Nanjing, China), which was set 12.5 Hz. The total capturing time was about 40 s. The camera-object and laser-object distances were set 315 mm and 640 mm, respectively. The incident angle was θ ≈ 30°. Image exposure time, gain and brightness of the CCD camera were optimized experimentally, in order to avoid overexposed pixels. The system and image acquisition parameters were kept constant during the experiment.

### 2.3. Data Processing Methods

#### 2.3.1. Visible Imaging

Two ROIs (65 × 65 pixels) within one image were selected to represent sound skin (SS) and injured skin (IS) (shown in Figure 1). Color and texture features were extracted from ROI images and implemented in LS-SVM to discriminate SS and IS. Color features consisted of mean values and standard deviations of three components of RGB. Texture features were separately obtained by GLCM [24] (Gray Level Co-occurrence Matrix), Gabor filter [25] and DT-CWT [26] (Dual-Tree Complex Wavelet Transform) in this paper. Detailed features used in visible imaging are listed in Table 1.

#### 2.3.2. Biospeckle Imaging

**Figure 4:**
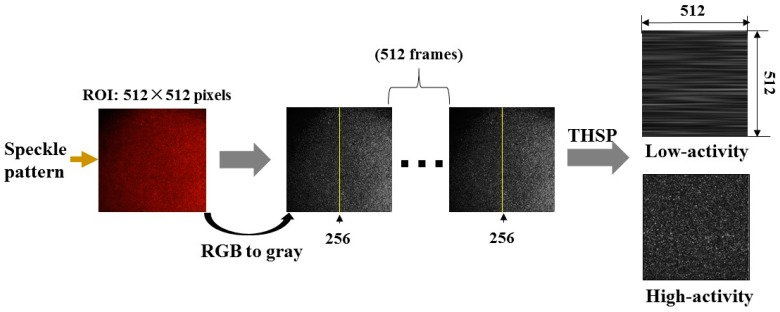
Flow chart of THSP formation (THSP: Time History of the Speckle Pattern).

Dynamic speckle (biospeckle) is demonstrated by a temporal sequence of images, and BA can be computed from these sequential images by various methods. The THSP-based IM (Time History of the Speckle Pattern-based Inertia Moment) method [27] was used to quantitatively assess BA in this paper. The THSP [28] is a matrix composed of a number of successive images. The THSP is created using the same column extracted from each image and placed side by side. The set of columns is arranged sequentially in chronological order. The width of the THSP is equal to the number of images used and represents the time scale of BA. The formation of THSP is illustrated in Figure 4.

IM reflects reliable information on the bio-activity of the living tissue. The first step to obtain BA is to create a matrix named COM defined as:

(1) $$COM=[N_{ij}]$$

This matrix is formed by the number of occurrences, represented by *N*, that a certain intensity *i*, is followed immediately by an intensity value *j*. When the intensity does not change, the only non-zero values of this matrix belong to its principal diagonal. As the sample shows activity, intensity values change in time and the number N outside the diagonal increases, so the associated COM is more spread.

In order to obtain a quantitative measure from this matrix, it is necessary to normalize it. This is done by dividing each row of the matrix by the number of times that the first grey level appeared.

(2) $$M_{ij}=\frac{N_{ij}}{\sum_{j}N_{ij}}$$

A measurement of the spread of the values around the principal diagonal can be constructed as the sum of the matrix values times its squared row distance to the principal diagonal. This is a particular second-order moment called the IM of the matrix with respect to its principal diagonal in the row direction.

(3) $$IM=\sum_{ij}M_{ij}{\left(i-j\right)}^{2}$$

where *M_ij_* is the COM adequately normalized. The occurrences in the diagonal do not contribute to increasing the IM value, while far away M entries add their more heavily weighted values. 

Finally, BA is assigned by IM,

(4) BA=IM

Therefore, BA is greater for samples with high activity and vise versa. It is treated as the feature to be introduced into the classifier of BLR to conduct classification.

### 2.4. Statistical Analysis

ROIs obtained from visible imaging were hand-selected from original RGB images, according to Figure 1. The size of ROI in biospeckle imaging was configured in our self-developed program on the platform of Visual Studio 2012 (Microsoft Corp., Redmond, WA, USA). In our study, ROIs with 512 × 512 pixels were cropped from original images (1600 × 1200 pixels). The size of ROIs was adjustable and kept constant during experiment. It is important to make sure the target skin (IS or SS) covers the ROI area. The 256th column in ROI images was selected to form THSP (shown in Figure 4). The extraction of pixel- and region-wise features in visible imaging and biospeckle imaging, along with LS-SVM classifications, were conducted using Matlab R2014a (MathWorks, Inc., Natick, MA, USA). BLR and ANOVA were employed on the platform of SPSS Statistics 20 (IBM Corp., Armonk, NY, USA).

## 3. Results

### 3.1. Experiment Results Obtained from Visible Imaging

**Figure 5:**
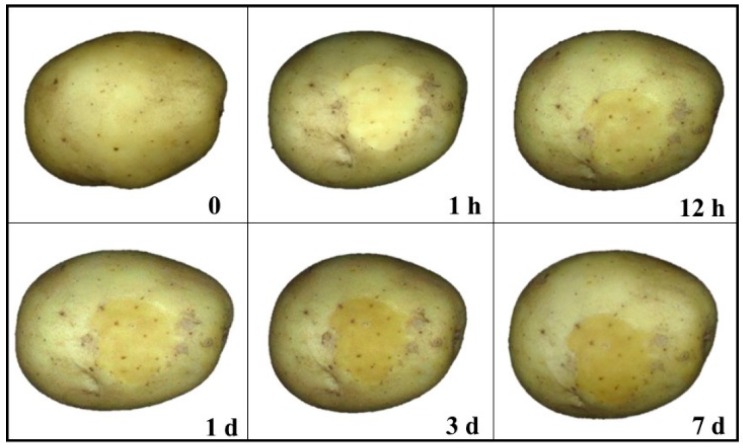
RGB images of potato tubers before and after skinning injury (0 indicates tubers before skinning injury).

Representative RGB images of sound and skinning injured tubers after storing for different time are shown in Figure 5. Generally speaking, contrast between SS and IS enhanced with the elapse of time (specifically shown in Table 2). The contrast was defined as Con, which was the difference of average gray value between SS and IS within ROIs. Con was negative in 1 h, because fresh injured area contained overflow water and reflect more light. One kind of protective film was then formed on skinning injured area after a period of time, probably because of the development of wound-induced suberization. It is also worth noting that the distribution of lenticels (dark spots shown in Figure 5) scattering in injured areas varied within seven days, which may be resulted from the meristematic action of the phellogen and periderm suberization [29,30]. The appearance of variation in regard to the color and texture features may lay the foundation for discrimination of SS and IS.

**Figure 6:**
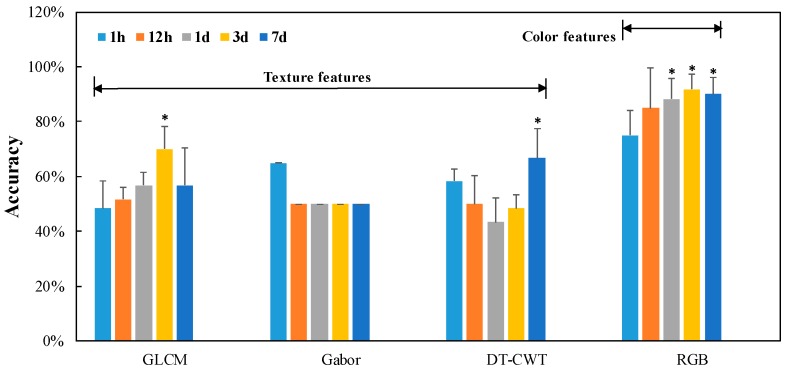
Classification results based on color and texture features, where * indicates significant difference (*p* < 0.05) compared with 1 h.

In visible imaging, ROI images were obtained on skinning injured tubers, representing SS and IS, respectively. Color and texture features (specifically shown in Table 1) were then extracted based on ROI images and implemented into LS-SVM. Distribution ratio between training set and prediction set was 2:1. Classification results are shown in Figure 6.

According to Figure 6, texture features applying in LS-SVM seemed not an appropriate way to classify SS and IS, with the highest prediction accuracy of 70%, regardless of the storage time after skinning injury and texture extracting methods. The average classification accuracies for different storage time with three methods were 56.67%, 53% and 53.33%, respectively. Color features behaved satisfying performance in discrimination of SS and IS, with the average prediction accuracy of 80% for injured potatoes storing within 1 d and 90% after storing for no less than 1 d. Classification results during different storage time were compared with that of 1 h. As can be seen, color features performed much better for injured skin with no less than 1 d (*p* < 0.05). Classification results based on combined features were not presented here, since the addition of texture features did not improve the results obviously.

### 3.2. Experiment Results Obtained from Biospeckle Imaging

**Figure 7:**
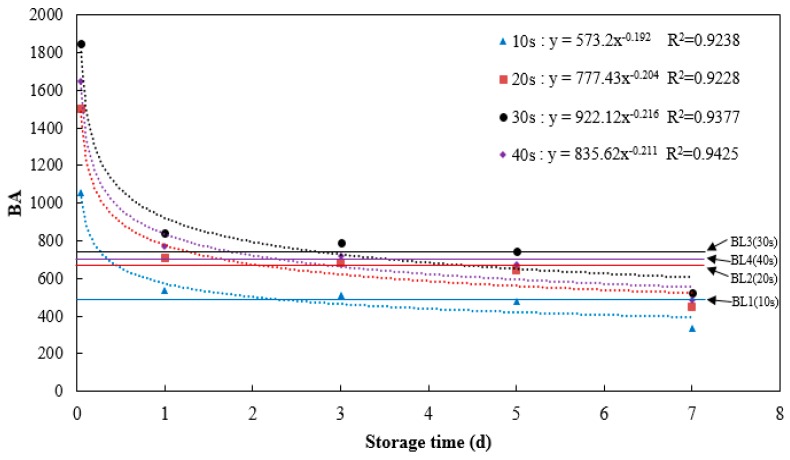
BA variation with time and comparison among different time spans.

Time dependency of BA variation calculated by speckle patterns within 40 s (512 frames) is shown as a purple line in Figure 7. Note that dots on the line correspond to 1 h, 1 d, 3 d, 5 d and 7 d. The four horizontal baselines (BL1-BL4) represent BA value of sound skin. It is understandable that these four lines showed different BA values, due to the interference of exterior factors and Brownian movement. As can be seen, BA in 1 h showed much higher values, implying that particle movement became more violent during this time. On the whole, BA increased dramatically for the first hour after skinning injury, then suddenly decreased through the next seven days till reach the stabilization. As shown in Table 3, BA in 1 h was significantly higher (*p* < 0.05).

However, this method is time-consuming if taking all speckle patterns into consideration. To increase image capturing and processing efficiency, BA values calculated by frames within 10 s, 20 s, 30 s and 40 s were compared (shown in Figure 7). Frames within 10 s, 20 s and 30 s were all intercepted from original 512 frames. As can be seen, time dependencies of BA for different time spans were consistent, indicating that speckle patterns needed for charactering BA can be possibly reduced to 125 frames from 512 frames. Correlation analyses were conducted between 10 s and all other time spans (shown in Table 4). As can be seen, BA calculated within 10 s is significantly and highly correlated with that of other time spans (*p* < 0.05), which verified the feasibility of capturing and processing speckle patterns within 10 s instead of 40 s. Additionally, BA values calculated by frames within 30 s showed the maximum compared with other time spans. This may be caused by the presence of exterior noise, which can bring about interference and result in an increase in IM values [27].

**Figure 8:**
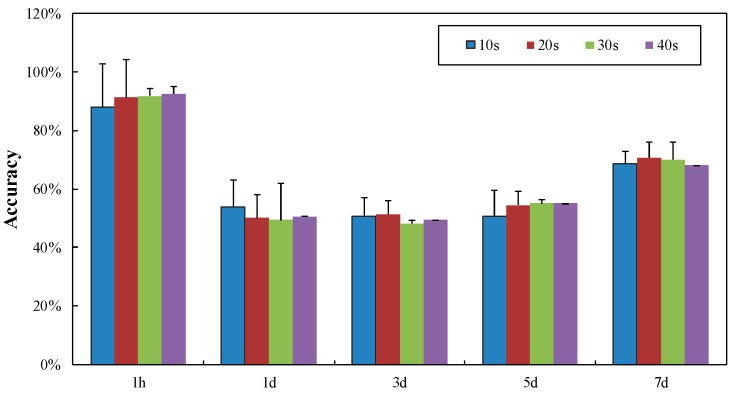
Classification of sound skin and injured skin by BLR.

BA calculated by speckle patterns within 10 s, 20 s, 30 s and 40 s were employed as the features into BLR to discriminate injured skin after 1 h, 1 d, 3 d, 5 d and 7 d from sound skin, as illustrated in Figure 8. It can be seen that classification performances of BLR with BA calculated within different time spans were similar, demonstrating the feasibility of using 10 s as the capture time of speckle patterns instead of 40 s. Classification result within 10 s was acceptable for sound skin and injured skin storing for 1 h, with the accuracy of 88.10%. In addition, classification performance for injured skin within 1 h was much better (*p* < 0.05) compared with other storage periods, and the classification accuracy decreased afterwards.

### 3.3. Comparison of Classification Results Based on Visible and Biospeckle Imaging

As can be seen from Figure 6 and Figure 8, color features derived from visible imaging produced satisfying results for injured potatoes stored no less than one day, with the average classification accuracy of 90%. Classification result was still acceptable (88.1%) for sound skin and injured skin stored less than one day, especially considering the great improvement of efficiency. Time effect (1 h and 1 d) on classification performance was implemented using ANOVA (shown in Table 5). It can be concluded that visible imaging is suitable for discriminating injured skin with no less than 1 d, while biospeckle imaging is regarded as effective in differentiating sound skin and injured skin within 1 d.

## 4. Discussions

**Figure 9:**
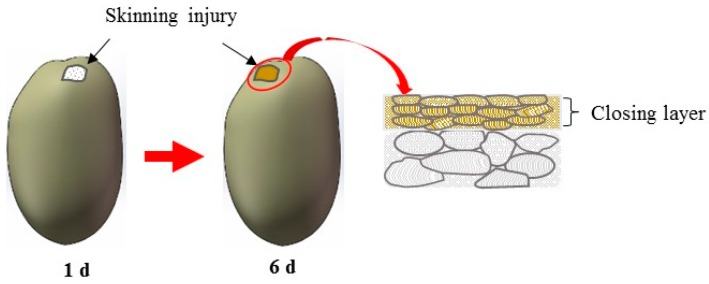
Schematic diagram of closing layer formation after skinning injury.

Wound-induced suberization begins to establish after skinning injury. According to Lulai [4], tuber tissue suberization involves two stages: (1) “closing layer” is formed whereby the walls of existing cells at the wound site suberize, which is often referred to as “primary suberization”; and (2) “wound periderm” whereby files of new cells are formed and suberized below the closing layer, which is often referred to as “secondary suberization”. Schematic diagram of closing layer formation was shown in Figure 9. Suberized rapidly neighboring the wound surface, closing layer serves to provide the initial protective barrier for the injury [31]. According to Lulai et al. [22], rapid wound-induced increase in Polyamines biosynthesis was observed during closing layer formation. Within 6 h of wounding, increases in the in vitro activities of enzymes (arginine and ornithine decarboxylase) and expression of related genes were observed. Previous study also indicated that closing layer development completes at about six days after injury [31,32]. The tendency of BA after injury shown in Figure 7 approximately conforms to the description above, indicating the feasibility of biospeckle imaging in characterizing vitality.

Within one day, physiological and biochemical reactions under wounded areas are especially fierce, particle movements become more active. After one day, closing layer on wounded area becomes established, implying the start of new periderm formation. Though secondary suberization is also involved [4], biospeckle imaging is more likely to reflect surface or sub-surface information. The similarity of skin and flesh colors may confuse the classification performance within a short time, whereas the surface color of the injured skin becomes darker with the elapse of time due to deepening extent of oxidation for sufficient exposure to exterior environment, which guaranteed the feasibility of skinning injury recognition using color features during late stage. Texture features seem not very suitable in classifying SS and IS because of the complex and irregular textural changes, considering the suberization, the variation of lenticels and unpredictable environmental influences. Meanwhile, the scale of ROI images was limited, which may not represent the whole textural changes within injured areas. Biospeckle activity may result from processes related with movement of the scattering centers in the tissue, such as cytoplasmic streaming, organelle movement, cell growth and division during maturation and biochemical reactions [33]. As stated by Zdunek [34], in organs with a high pigment content or in the final stages of development biospeckle activity is relatively low. There is no research connected with color change effect on biospeckle activity up to now. Theoretically, particle movement and biochemical reactions caused by color change may affect biospeckle activity. However, how these two factors are related is still unknown.

As mentioned above, these two techniques are complementary to each other in recognition of skinning injury. Both configuration systems are low-cost, yet visible imaging system is much more mature and stable. Biospeckle imaging system is more vulnerable to exterior fluctuations, and all optical elements including laser, beam expander and polarizing film should be assembled precisely to obtain clear images on CCD sensor. In regard to time cost, visible imaging generated characteristic features more quickly, though great improvement of efficiency can be achieved considering BA computed within 10 s instead of 40 s. Furthermore, visible imaging technique has been utilized in commercial production, while biospeckle imaging technique is still restrained in lab. Nevertheless, biospeckle imaging has great potential in characterizing surface and subsurface information, which has been proved previously in our team [19].

## 5. Conclusions 

This paper presents the discrimination of SS and IS using CCD-based visible and biospeckle imaging techniques. For visible imaging, color and texture features were extracted and introduced into the classifier of LS-SVM. BA calculated by speckle patterns based on biospeckle imaging was regarded as the particular feature, and employed into another classifier of BLR. Results showed that color features performed better than texture features, especially for injured potatoes after storing for no less than 1 d, with the average classification accuracy of 90%. Complementarily, BA was a feasible feature in BLR when discriminating SS and IS within 1 d, with the classification accuracy of 88.10%. In addition, great efficiency improvement can be realized via capturing and processing speckle patterns within 10 s instead of 40 s.

In the future, the selection of ROI images shall be more subjective, to make sure the SS and IS are more representative and consistent. Other features, like fractal dimensions, can be further evaluated. BA calculated by THSP-based IM was not a perfect method to characterize potato vitality, so other quantitative methods could be assessed in the future. Besides, acquisition time of speckle patterns still has room to be reduced to improve efficiency further.

## Figures and Tables

**Table 1:** Color and texture features used in visible imaging.

Category		Number of Features	Description
Color	RGB	6	Mean values and standard deviations of three color channels of RGB
Texture	GLCM	8	Mean values and standard deviations of ASM (Angular Second Moment), ENT (Entropy), INE (Inertia) and COR (Correlation)
	Gabor	108	Twelve filters with 3 scales and 4 orientations, with one image divided into 3 × 3 image blocks
	DT-CWT	12	Real and imaginary images at approximately ±15°, ±45° and ±75°, respectively

**Table 2:** Variation of image contrast with the elapse of time.

	1 h	12 h	1 d	3 d	7 d
Con *	−13.064 ± 5.429 ^d^	18.832 ± 3.965 ^c^	30.943 ± 4.464 ^a,b^	37.436 ± 4.882 ^a^	28.983 ± 3.249 ^b^

* Indicates significant differences when with different letters (a, b, c, d) (*p* < 0.05).

**Table 3:** BA variation with the elapse of time.

	0	1 h	1 d	3 d	5 d	7 d
Mean value *	2421.08 ± 115.323 ^b,c^	3903.28 ± 390.157 ^a^	2771.88 ± 82.416 ^b^	2647.68 ± 114.337 ^b^	2753.90 ± 129.702 ^b^	2196.21 ± 192.280 ^c^

* Indicates significant differences when with different letters (a, b, c) (*p* < 0.05).

**Table 4:** Correlation analysis between 10 s and other time spans.

	0	1 h	1 d	3 d	5 d	7 d
10 s	1	1	1	1	1	1
20 s	0.968 *	0.931 *	0.926 *	0.956 *	0.963 *	0.960 *
30 s	0.966 *	0.915 *	0.897 *	0.957 *	0.956 *	0.938 *
40 s	0.965 *	0.928 *	0.921 *	0.949 *	0.959 *	0.948 *

* Indicates significant correlation (*p* < 0.05).

**Table 5:** ANOVA between 1 h and 1 d on two techniques.

**Statistics**	**Visible Imaging**		**Biospeckle Imaging**	
	1 h	1 d	1 h	1 d
**Mean Value**	75%	88.33%	88.1%	53.8%
**ANOVA between 1 h and 1 d**				
***F*-Value**	8.930		19.044	
**Level of Significance *p***	0.024		0.005	

## Abbreviations

CCD	Charge Coupled Device
ROI	Region of Interest
BA	Biospeckle Activity
DT-CWT	Dual-tree Complex Wavelet Transform
GLCM	Gray Level Co-occurrence Matrix
THSP	Time History of the Speckle Pattern
IM	Inertia Moment
LS-SVM	Least Square Support Vector Machine
BLR	Binary Logistic Regression
